# Protein Localization with Flexible DNA or RNA

**DOI:** 10.1371/journal.pone.0029218

**Published:** 2012-02-10

**Authors:** Sebastian Bernhardsson, Namiko Mitarai, Kim Sneppen

**Affiliations:** 1 Niels Bohr institute, University of Copenhagen, Copenhagen, Denmark; 2 FOI, Swedish Defence Research Agency, Tumba, Sweden; University of Hyderabad, India

## Abstract

Localization of activity is ubiquitous in life, and also within sub-cellular compartments. Localization provides potential advantages as different proteins involved in the same cellular process may supplement each other on a fast timescale. It might also prevent proteins from being active in other regions of the cell. However localization is at odds with the spreading of unbound molecules by diffusion. We model the cost and gain for specific enzyme activity using localization strategies based on binding to sites of intermediate specificity. While such bindings in themselves decrease the activity of the protein on its target site, they may increase protein activity if stochastic motion allows the acting protein to touch both the intermediate binding site and the specific site simultaneously. We discuss this strategy in view of recent suggestions on long non-coding RNA as a facilitator of localized activity of chromatin modifiers.

## Introduction

Molecules in the cell that work together are often co-localized. For example, transcription factors can act on promoters even when binding on distant operator sites. Other examples of localization is transcription in certain transcription factories [1], [2], and DNA repair proteins which tend to localize on DNA around sites of DNA damages [3]. Recently also long non-coding RNA (lncRNA) have been found to play a role in regulating enzymatic activities in *cis*, [4]–[10]. Even within the cytoplasm of the small prokaryotic cell there is localization, for example in form of co-localization of transcription and translation [11], and localization of a number of proteins around the DNA replication fork [12].

The function of co-localization is not yet fully understood, but one can think of several possibilities. Firstly, efficient localization of active proteins close to the target cite reduces non-specific reactions. If a protein act while it is tethered to the mRNA it is translated from, it will facilitate regulation that only act in *cis* thereby opening for more fine tuned regulatory systems. For example, the antiterminator Q in phage 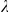 does preferably act on its own genome [13], thereby preventing other related phages from hijacking a lytic decision. Another advantage of reducing non-specific reactions, may be to prevent collateral damage by reactions that are only designed to deal with extreme situations, such as DNA-damage. Here we focus on increased geographical specificity as a way to localize activity at the target by shortening the time for a protein to locate the target.

**Figure 1:**
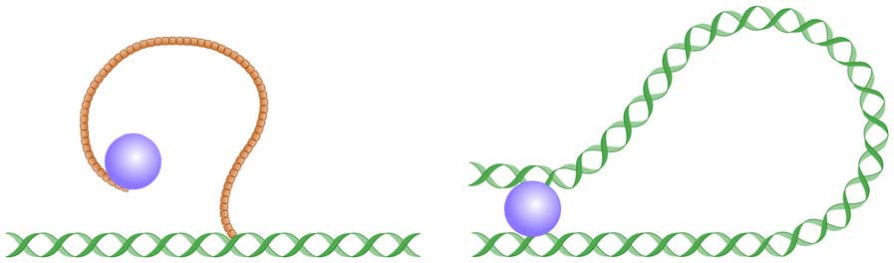
Localization of a protein by using respectively a lncRNA or the looping abilities of DNA. In both cases the Brownian motion of the protein will be restricted, increasing the local concentration of the protein at target site by an amount given by the J-factor, see review by [21].

The simplest, and perhaps the only way to realize localization is to place intermediate binding sites (IBSs) around the target sites. However, this is in itself not enough. Even though a locally high density of such sites will increase local concentration of the protein, they may not increase the activity at a specific target site. That is because the proteins spend a lot of time by binding at the IBSs but not at the target. In order to gain activity, the protein need to be able to access the target while it is still bound to the IBS. And furthermore, the gain in protein activity will be closely linked to the time it takes the protein bound to the IBS to diffuse and localize the target. In Fig. 1 we illustrate a protein bound to intermediate binding sites (IBS) on respective a lncRNA or a DNA, and indicate that it thereby gain better access to a specific site on the DNA. In this paper we explore efficiency of target localization as function of properties of the IBS.

## Methods

We here explore theoretically how activity can be increased by introducing a polymer near the target site, a polymer that can bind the protein and thereby localize its search. The polymer is supposed to be attached at the target, and to have an intermediate binding site (IBS) where the protein can bind. Accordingly, the probability distribution for the IBS to be at a distance of 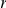 from the target is approximated by the Gaussian distribution
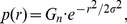
(1)where the parameter 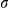 is associated to the length of the polymer and 
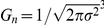
 is the normalization, which represent the probability density that the IBS is present at position 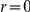.

When the protein is not bound to the IBS, it performs a free diffusion with probability to be trapped by an IBS given by 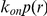, where 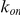 is the number of IBSs present (i.e. several polymers or several IBSs on a single polymer). Similarly, the protein unbinds from an IBS with rate 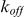. When trapped, the protein makes a biased random walk reflecting motion inside a harmonic potential with diffusion constant 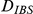. It therefore moves slower with a step length reduced by 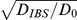 relative to the free motion. When the protein is within the target radius, 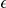, the target is supposed to be found, independently whether the protein is bound to an IBS or is free.

The calculations were performed as Monte-Carlo simulations for a single particle moving in a box of size 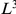 and with reflecting boundary conditions. The simulation is performed using discrete time and direction (x-, y- and z-direction). The step length is given by the velocity 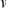 (the simulation time step is unity). For free movement we use 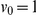, and thus 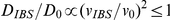, since 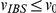. The bias is introduced by multiplying the probability for movements where radius 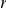 is increased by a factor 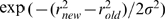.

The particle starts from a random position, and during one time step the particle can either move in space, bind/unbind from the IBS or bind to the target, with equal probability (a more detailed description of the algorithm is given in the supplementary Information S1, section A). One realization of the simulation ends when the target is found. All results presented following are averaged over 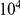 realizations.

## Results

**Figure 2:**
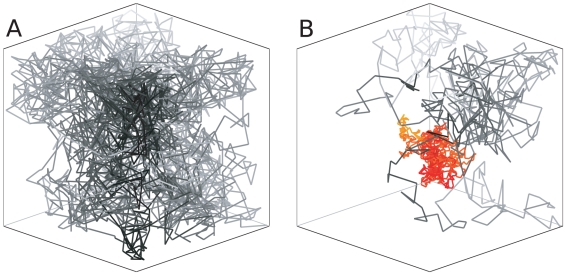
Typical trajectory of target search. Model cell of width 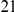 in simulation units, with one target site located in center of diameter 1. A) Trajectory of a randomly released protein until the target is reached. B) As in A) but with possibility to bind to intermediate binding sites (IBS) with probability 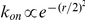 and to release from this site with low 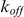. When bound to the IBS the protein moves stochastically with 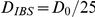 while sampling the localized distribution 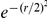 (colored trajectory).

The environment inside a cell is noisy, and a free protein should search a potential target through diffusion as described by a Brownian random walk. Fig. 2A shows such a trajectory of a protein as it searches a target in the center of a 3-dimensional cube, that represent an idealized cellular compartment. The associated search time is given by [14]

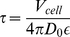
(2)where 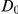 is the diffusion constant (reported for GFP in mammalian cells to range from 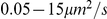, depending on compartment and the type of protein it is fused to [15], [16], whereas the diffusion for GFP in *E.coli* was 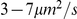 [17]). 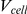 is the volume of the available cellular compartment and 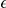 the radius of the target. In *E.coli* where 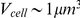, the search time for a single protein would be of order 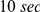 [18], [19], whereas in an Eukaryotic cell compartment should be of the order 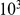 times larger just due to the larger volume. For LacI in *E.coli* the search time for the single protein was found to be order of 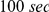 [18], reflecting additional time wasted on non-specific DNA far from the target site.

When a protein is trapped by an IBS, it can stay in the vicinity of the target and thus contribute to an increase in density. For a confining polymer, this increased density is called the J-factor, see [20], [21]. However, the increase of the apparent density itself does not increase its activity at the target, because the IBS will also limit the rate at which it samples the space around it. The focus of this paper is to address this interplay between increased local concentration and decreased local diffusion.

While the free protein diffuses with a relatively large diffusion coefficient 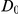, the IBS:protein complex diffuses with a diffusion coefficient 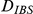 with a drift consistent with the movement of a long polymer confined in one end at the target site. We take this into account in our model by assuming that the protein bound to the IBS moves as a particle trapped in a harmonic potential, and the protein jumps in and out of the potential with rates given by the probability to encounter one IBS times the number of IBS's, and 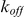, respectively. Moreover, 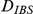 is smaller than the free diffusion 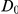, because it is more difficult to diffuse for a larger object. Further details of our real time Monte-Carlo simulations is given in the method section. Examples of trajectories for free diffusion and with IBS's present where 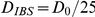 and 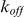 is very low is given in Fig. 2A and B. If a protein is assumed to diffuse with 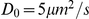 in a cell of diameter 1 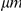, then one unit of length in our 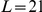 simulation correspond to 0.05 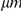 and thus one time step in the simulation 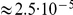 seconds. This means that 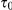 (time to find the target by free diffusion) from Fig. 2A is around 1.6 seconds, see eq. (2). (Note that this relatively short time is due to the relatively large target size of around 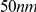, and reducing the target radius by a factor of 10 will increase the search time by the same amount).

**Figure 3:**
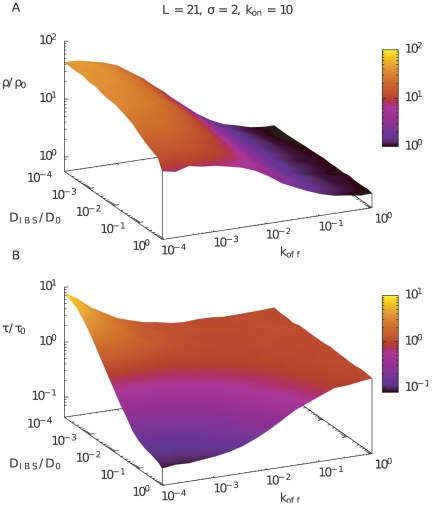
Average density and average search time. A) Average density within distance 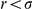 from the target of radius 0.5, for 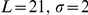 and 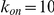 as a function of relative diffusion coefficient (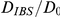) and affinity to IBS (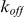). Notice that this density includes the freely diffusing proteins that in particularly contribute when 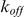 is large. B) Average time to reach the target for the same conditions as in A. 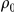 and 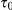 is the density and time in the case of free diffusion without IBSs.

Fig. 3 presents the main results of our simulations, showing the average density and the average search time for a protein that is released randomly in the cell, and is followed until it reaches the target in the center of the cell. Both density and time is measured relative to the case for free diffusion, 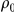 and 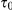 respectively. From Fig. 3A we see that the density around the target easily becomes many folds larger than the background. Panel B show that the increased density of active proteins around the target sometimes, but not always, can give rise to an increased activity at the target. In fact if the diffusion of the IBSs, 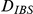, is very slow, the protein spends most of the time in the vicinity of the target but rarely reaches it. In the limit of no relative movement between IBSs and the target, the IBSs act as passive sinks for the target location, and the associated activity will drop towards zero.

Figure 3B shows a 10 fold decrease in search time for low 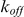 and 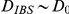, compared to free diffusion. Notice that the reduction in search time will be significant even when 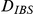 is reduced substantially below the free diffusion constant 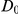, provided that the reacting protein remain tightly associated to the IBS (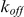 is low).

The detailed profiles shown in Fig. 3 depends on both cell volume and size of the region dominated by the IBSs. For small 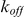 the search time can be estimated by adding the time to find first an IBS, and then the time to find the target in a volume accessed by the IBS. Thus the total search time is estimated by repeated use of the time needed for diffusion limited search (eq. (2)):
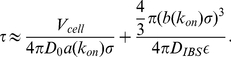
(3)The time to be captured by the IBS is estimated by the first term in eq. (3). Here 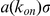 is the average distance from the target where the protein is captured by the IBS, a distance that naturally will depend on the on-rate, 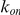. The second term in eq. (3) represents the time to find the target after being captured by the IBS. In our simplified equation we assume that after the protein is captured, it rapidly is dragged into a region of radius 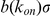 where the potential is so flat that the drift can be ignored. In this region the search is then approximated by an unbiased random walk with diffusion coefficient 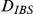. Naturally 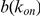 should be only weakly dependent on 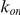, and in fact be of order 1, reflecting a flat potential of a width given by 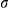.

**Figure 4:**
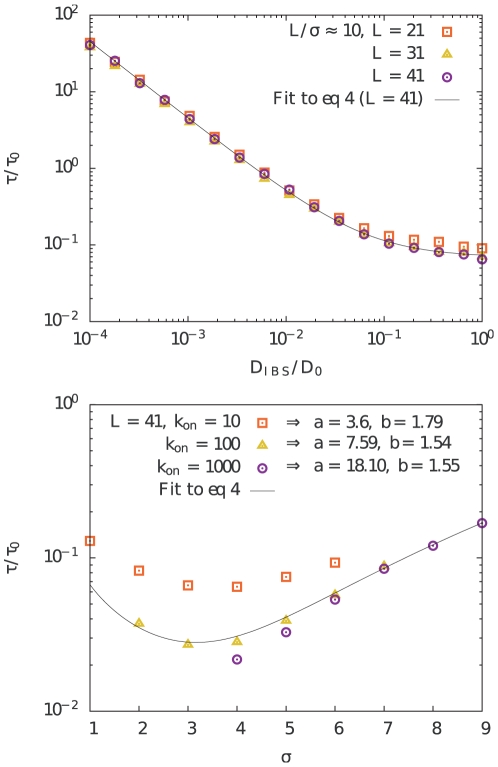
Parameter dependences of the search time. Relative search time, 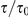, as a function of relative diffusion constant, 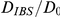 (upper panel), and the width of the Gaussian distribution, 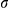 (lower panel). In the upper panel 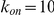 and the ratio 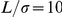 is kept roughly constant. The solid line is a fit to Eq. 4 with fitting parameters 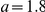 and 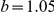. In the lower panel 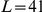 and 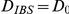. The solid line is a fit to Eq. 4 for the case 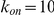, and the values of 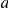 and 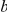 shown in the legend are the corresponding fitting parameters for each case.

The search time relative to free diffusion (eq. (2)) can then be written as
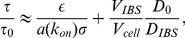
(4)where 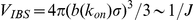, with 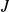 being the J-factor in case of a confining polymer [20], [21]. For small 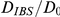 the search time 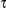 is dominated by the second term, and 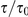 becomes 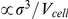. Thus, in this limit, modulation of search time by the IBS depends only on the ratio 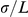. The validity of our approximate equation for the search time eq. (4) is confirmed numerically in Fig. 4a where 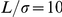 and 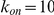. The figure also demonstrates that the curves for different 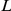 collapses on top of each other for small 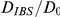 provided rescaling into units of the cell size. When 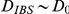 then 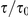 decreases as 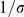, as expected from the first term of eq. (4).

The solid line in the figure represents a fit to eq. (4). It is important to note that when the target size, 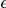, is decreased, keeping 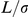 fixed and 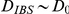, the gain in search time increases linearly until the second term starts dominating. At this point the gain and loss in search time is determined by the ratios of IBS volume to cell volume.

Another interesting property is that the search time has a broad minimum as a function of 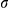 for fixed 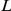 (Fig. 4b). That is, the gain in search time is basically the same when 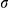 varies between all the way from 4% to 24% of the cell radius. For small 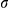 (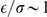 and 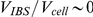) the IBS ceases to play a role and finding the IBS takes as long time as finding the target by free diffusion. On the other hand, a very large 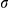 (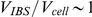 and 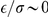) implies that the IBS is essentially non-specific, and finding the target when trapped takes as long as when free, given that 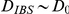. In both these cases 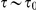, while a mixed situation can give 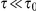.

## Discussion

This paper analyzed a simple strategy for localizing activity inside the living cell, using a simple Gaussian confinement model (harmonic potential). The analysis was inspired both by the multiple observations of localized activity, as well as by recent proposals on possible roles of long RNA transcripts in regulating nucleosome modifying factors in *cis* [4]. Although our formalism, where we model the intermediate binding site by a Brownian walk confined in a harmonic potential, was designed to mimic the end to end distance in a polymer like the mRNA, it may as well apply to other flexible structures such as DNA or the nuclear matrix [22]–[26]. The main feature that required is specificity in the protein-IBS binding *and* a flexible IBS, such that the protein indeed get confined in some particular region that facilitate contact to the target site.

There are a few specific lessons that we would like to emphasize: First, introducing additional binding sites close to target-site of activity of a given protein does not necessarily imply an increased activity at the target (this is also true for the equilibrium case which is explored in the supplementary Information S1 section B). In particular, if the additional binding site is rigid, or does not allow for direct transfer of the protein to the target, then additional binding sites in fact lowers the activity of the protein. In order for an IBS to catalyze the activity (lower the search time), the IBS must also be able to move relative to the target. The gain will then depend on the relative size of the cell, the volume spanned by the IBSs and the target, as well as the diffusion coefficient of the relative movement between the protein while it is bound to the IBS, and the target.

Under all conditions, the presence of IBS somewhere in the cell, will reduce the likelihood that the protein in question is active elsewhere. Thereby IBS can in fact also be used to reduce “collateral” damage of for example DNA-repair proteins. The repression of such collateral damage will be at least as large as indicated by the 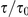 ratio in Fig. 3B, and in fact substantially larger if the active protein is released closed to the target and is subsequently is inactivated or degraded on a timescale that is shorter than the time it takes the protein to escape the IBS (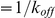).

Overall, the gain of the proposed search strategy involving binding to a long non-coding RNA (lncRNA) could be as big as the volume explored by the lncRNA divided by total volume of cell nucleus. For a lncRNA that localize the protein to a region of diameter 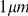 around the target in a mammalian cell nucleus (diameter 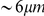), the gain of activity should maximally be 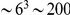.

Finally, we would like to address the importance of experimental verification of the present results to quantitatively understand the function of localization. Our results predict that 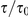 will be a function of 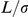 as long as 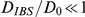 and the off-rate is small, which can be tested by in vitro experiment by for example using single-molecule fluorescence resonance energy transfer (smFRET) [27] ; the key point of the experiment will be to distinguish the binding to the target and the binding to the IBSs. To verify our results, the binding kinetics needs to be measured with various volume of the container (which determines 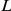) and the length of the polymer (which determines 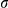). In addition, such a setup would allow for varying 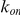 through changing the number of binding sites on the polymer, while 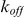 will be determined by the strength of the available binding sites on the polymer.

## Supporting Information

Information S1 Supplement A: Simulation algorithm. Supplement B: Equilibrium considerations.
(PDF)
