# Supplementary material for: Protein Localization with Flexible DNA or RNA
Source: PLoS One. 2012 Feb 10;7(2):e29218. doi: 10.1371/journal.pone.0029218 (PMC3277508; doi:10.1371/journal.pone.0029218)
Supplement: Information S1 — Supplement A: Simulation algorithm. Supplement B: Equilibrium considerations. (PDF) [file pone.0029218.s001.pdf]

Protein localization with flexible DNA or RNA
(Supplementary material)

Sebastian Bernhardsson[1]
Niels Bohr institute, CMOL
University of Copenhagen, Denmark

Namiko Mitarai
Niels Bohr institute, CMOL
University of Copenhagen, Denmark

Kim Sneppen
Niels Bohr institute, CMOL
University of Copenhagen, Denmark

November 30, 2011

[1]Corresponding author. Address: Niels Bohr institute, CMOL University of Copenhagen, Blegdamsvej 17, 2100 Copenhagen, Denmark E-mail: sebbeb@nbi.dk

Supplement A: Simulation algorithm

In order to obtain detailed balance in equilibrium, every simulation step is devided up in to the possible moves between the different states, all with equal probability (1/3 for exclusive binding and 1/4 for inclusive binding, see Supplement C). The algorithm below describes in regular type the simulation for the case of exclusive binding, where the points in italic type corresponds to the additional steps needed to simulate the case of inclusive binding.

If not at the target:

1. Move in space:
   a) If the particle is free it is moved a distance $v_0 = 1$, along one of the 6 directions on the lattice.
   b) If the particle is bound to an IBS at position $r = (x, y, z)$, it is moved a distance $v_{IBS} \leq v_0$, to position r_{new} along one of the 6 directions on the lattice with a probability given by the Boltzmann factor $e^{-(r_{new}-r)^2/2\sigma^2}$.

2. Move between IBS and free:
   a) If the particle is free at position $r = (x, y, z)$ it is moved to a state bound to the IBS with probability $\frac{n_{on}}{\sqrt{2\pi\sigma^2}^3} e^{-(r-r_0)^2/2\sigma^2}$ where $r_0 = (x_0, y_0, z_0)$ is position of the target.
   b) If the particle is bound to the IBS it is moved to the free state with probability k_{off} independently on its position.

3. Move to target:
   a) If the particle is free and at a position $|r - r_0| < \epsilon/2$ it binds to the target with probability 1
   b) If the particle is bound to an IBS and at a position $|r - r_0| < \epsilon/2$ it binds to the target with probability 1.

4. *Move to target + IBS:)*
   a) If the particle is free and at position $|r - r_0| < \epsilon/2$, it binds to both target and IBS with probability 1.
   b) If the particle is bound to an IBS and at position $|r - r_0| < \epsilon/2$, it binds to both target and IBS with probability 1.

If bound to the target:

1. Move in space:
   a) Nothing happens.
   b) If also bound to IBS nothing happens.

2. Move to Free:
   a) The particle is released from the target with probability δ.
   b) If also bound to IBS the particle releases from both with probability $\delta k_{off}\sqrt{2\pi\sigma^2}^3/n_{on}$.

3. Move to IBS:
   a) The particle is released from the target and bound to an IBS ($r = r_0$) with probability $\frac{n_{on}\delta}{k_{off}\sqrt{2\pi\sigma^2}^3}$.
   b) If also bound to IBS the particle is released from the target with probability δ.

4. *Move between target and target + IBS:*
   a) If the particle is only bound to the target, the particle also binds to an IBS ($r = r_0$) *with probability* $n_{on}/\sqrt{2\pi\sigma^2}^3$.
   b) If the particle is bound to both the target and an IBS, it releases from the IBS with probability k_{off}.

The algorithm for exclusive binding is used for the non-equilibrium case where a particle is released at a random position, but the simulation stops once the target has been found. As a consequence of the detailed balance requirement, 3 time-steps of our simulation will on average make a free particle diffuses one lattice point.

Supplement B: Equilibrium considerations

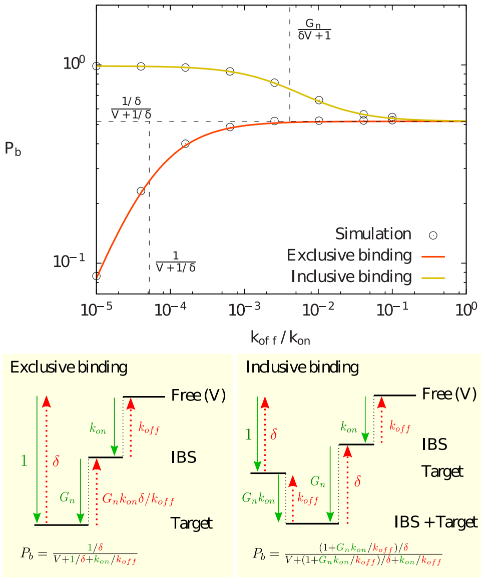

Figure S1: *Upper panel: Probability to bind to the target as a function of binding to intermediate binding sites. In equilibrium, diffusion dynamics becomes irrelevant, so we only show behaviour as function of binding affinity to the IBS. (k_{off}/k_{on}). Lower panel: Energy landscapes for the exclusive and inclusive binding, together with corresponding jumping rates. The probability to be bound to the target, P_b can then be found by the partion function method. G_n is the Gaussian normalization which equals the probability for an IBS to be located at the target ($r = 0$). The ratio of the off- and on-rate of the protein to the target is $1/\delta$.*

Throughout the paper we analyzed the non-equalibrium case where the particle was released randomly, and disappeared when reaching the target site. Here we instead consider a protein which is allowed repeatedly to bind and escape from the target, thereby establishing an equilibrium situation. In that limit, the activity probability to be at target can be calculated analytically, using the partition function method, see lower panel of Fig. S1.

In equilibrium, the proability to be at at the target site will be independent of whether the IBS is located around the target site or displaced away from it. Accordingly, the probability to be at the target site is decreasing as the protain spend more time in the state bound to an IBS by having smaller off-rate, k_{off}, and/or having larger over all on-rate, k_{on}. This is shown by the exclusive binding curve and equation in Fig. S1. The decrease reflect an

IBS that effectively work as a passive sink for the protein in question, also if the IBS is located just around the target site. A sink which will grow with increasing binding strength to the IBS.

The "passive sink" can be circumvented if the protein can bind *simultaneously* to the target and the IBS, which we refer to as the inclusive binding case in the figure. This require that the protein has two binding sites. In that case the probability to bind to the target site can then increase with the presense of IBSs, as described by the inclusive binding equations in Fig. S1. This inclusive binding case correspond to a DNA looping scenario, found to increase efficiency of transcription factors also for very long loops, for example phage λ (1).

References

[1] B. Revet, B. von Wilcken-Bergmann, H. Bessert, A. Barker, B. Muller-Hill, Four dimers of lambda repressor bound to two suitably spaced pairs of lambda operators form octamers and dna loops over large distances, Curr Biology 9 (3) (1999) 151–154.
